# A comparison of short- and long-term prognoses between cases with and without antenatal corticosteroid administration in late preterm delivery: a nationwide population-based study

**DOI:** 10.1186/s12884-024-06851-y

**Published:** 2025-02-12

**Authors:** Geum Joon Cho, Chan-Wook Park, Kyu-Dong Cho, Sungyeon Ha, Suk-Joo Choi, Min-Jeong Oh

**Affiliations:** 1 Department of Obstetrics and Gynecology, Korea University Guro Hospital, Korea University College of Medicine, 148, Gurodong-ro, Guro-gu, Seoul, 08308 Korea; 2 Department of Obstetrics and Gynecology, Seoul National University College of Medicine, Seoul, South Korea; 3 Big Data Department, National Health Insurance Service, Seoul, Gangwon-do Korea; 4 Graduate School of Statistics, Sungkyunkwan University, Seoul, Korea; 5 Department of Obstetrics and Gynecology, Samsung Medical Center, Sungkyunkwan University School of Medicine, Seoul, Korea

**Keywords:** Hypoglycemia, Late preterm, Neurodevelopmental outcome, Respiratory complication, Steroid

## Abstract

**Background:**

There is a paucity of information concerning the short- and long-term benefits and harm of antenatal corticosteroid administration and of expanded corticosteroid administration with dexamethasone in the late preterm period. Thus, we aimed to compare the effect on short-term respiratory complications, hypoglycemia, and long-term neurodevelopmental disorders in neonates born in the late preterm period between cases with and without corticosteroid administration and evaluate the difference in effects according to the type of corticosteroid administered.

**Methods:**

This retrospective observational cohort study included all women who had a singleton delivery in the late preterm period between January 2007 and December 2015. We extracted data from Korea National Health Insurance claims and National Health Screening Program for Infants and Children databases. Primary short-term outcomes were in the late preterm period. Concerning short-term effectiveness for respiratory morbidity, dexamethasone administration in the late preterm period was associated with respiratory complications and hypoglycemia in neonates. Long-term outcomes were neurodevelopmental disorders in infants/children observed at follow-up among all neonates until the end of 2018.

**Results:**

Of 57,963 women who delivered late preterm births during the study period, 1,255 (2.2%) had received antenatal corticosteroid administration in late preterm period. Dexamethasone administration was associated with a decreased risk of transient tachypnea (adjusted odds ratio [aOR] 0.66, 95% confidence interval [CI] 0.50–0.88) compared with no antenatal corticosteroid administration, but this effect was not observed in relation to betamethasone administration (aOR 0.69, 95% CI 0.42–1.14).

**Conclusions:**

Dexamethasone administration in late preterm infants was associated with a decreased risk of transient tachypnea compared with no corticosteroid administration but this effect was not observed with betamethasone administration. However, antenatal corticosteroid administration in the late preterm period did not lower the risk of other respiratory complications nor increase the risk of hypoglycemia, with no effect on neurodevelopment regardless of the type used.

## Background

The beneficial effect of antenatal corticosteroid (ACS) administration before 34 weeks of gestation has been well established [1, 2]. Guidelines have long recommended the routine use of a single course of corticosteroids for women at risk of preterm birth within 7 days between 24/0 weeks and 33/6 weeks [3–5]. This recommendation did not extend to women at risk of preterm birth after 34 weeks of gestation until May 2016, owing to an absence of reliable evidence concerning the effectiveness of ACS; however, late-preterm neonates are known to be at a high risk of mortality and morbidity due to conditions such as respiratory disorders [6–10]. 

Two small randomized trials evaluated the effect of betamethasone administration in the late preterm period on neonatal respiratory outcomes [11, 12]. However, both trials’ findings were inconclusive. The double-blind placebo-controlled randomized Antenatal Late Preterm Steroids (ALPS) trial reported that antenatal betamethasone administration in women expected to deliver in the late-preterm period decreased the rate of neonatal composite respiratory treatment, stillbirth, or neonatal death within 72 h post-delivery [13]. A recent meta-analysis of three trials, including the ALPS trial, reported similar findings [14]. Based on these results, current American College of Obstetricians and Gynecologists’ guidelines recommend a single course of betamethasone for pregnant women in the late-term period at risk of preterm birth within 7 days who have not previously been administered ACSs [4]. However, two other studies reported inconclusive results [15, 16], citing issues such as hypoglycemia of neonates and unknown neurodevelopmental outcomes of infants and children in cases treated with betamethasone [14]. 

ACS administration in the late preterm period may have the potential to adversely affect glycemic control in neonates and neurodevelopmental outcomes in infants/children, and no studies have reported evidence to support short-term (i.e., hypoglycemia) and long-term safety (i.e. neurodevelopment outcomes) for ACS administration in the late preterm period. Given this context, one study has suggested that indications for ACS administration should not be expanded to the late term period [17]. 

Moreover, it remains unclear whether ACS administration with dexamethasone in the late preterm period should be expanded. In clinical practice, two corticosteroids, namely, betamethasone and dexamethasone, are generally preferred for antenatal treatment to accelerate fetal organ maturation. In a Cochrane review of studies comparing antenatal betamethasone and dexamethasone, similar efficacy was observed between the two corticosteroids in relation to the risk of neonatal death, respiratory distress syndrome (RDS), neonatal intensive care unit (NICU) admission, bronchopulmonary dysplasia (BPD), periventricular leukomalacia, necrotizing enterocolitis (NEC), or retinopathy of prematurity [18]. Owing to inconsistent and limited data, American College of Obstetricians and Gynecologists guidelines suggest that one corticosteroid regimen cannot be recommended over the other [4]. However, most studies have evaluated the effectiveness of betamethasone administration in the late preterm period, with only a few studies involving small sample sizes having evaluated the effectiveness of dexamethasone administration in the late preterm period, with inconclusive results [16, 19–21]. To date, there is a paucity of information concerning the short- and long-term benefits and harm of ACS administration and of expanded ACS administration with dexamethasone in the late preterm period.

This nationwide population-based study aimed to compare the frequency of short-term neonatal respiratory complications and hypoglycemia and long-term neurodevelopmental disorders between cases with and without ACS administration, and between cases with betamethasone or with dexamethasone administration, in neonates born to mothers at risk of late preterm delivery.

## Methods

### Data characteristics

In Korea, 97% of the population is enrolled in the Korea National Health Insurance (KNHI) program. All claims information for these individuals is contained within the KNHI claims database. Almost all information concerning the prevalence of different diseases can be obtained from this centralized database, with the exception of procedures not covered by insurance.

As part of the KNHI system, children aged 4–80 months are eligible for a National Health-Screening Program for Infants and Children (NHSP-IC). An NHSP-IC is composed of seven consecutive health examinations based on age grouping. Health examinations 1–7 are performed at ages 4–9 months, 9–18 months, 18–30 months, 30–42 months, 42–54 months, 54–66 months, and 66–80 months, respectively. The NHSP-IC consists of history taking, physical examination, anthropometric measurements, screening for visual acuity, and questionnaires with anticipatory guidance. The NSHP-IC also includes the Korean Ages and Stages Questionnaire (K-ASQ) to screen for developmental delays between the 2nd and 7th health examinations.

### Dataset

The KNHI claims database is linked to data in relation to women’s offspring contained within the NHSP-IC database. Using the KNHI claims database, we identified all women who delivered singletons between January 1, 2007, and December 31, 2015 (*n* = 3,721,636). Women were excluded from analysis if their offspring did not undergo at least one of the seven consecutive NHSP-IC examinations or where there was missing data (*n* = 439,896). After excluding women who delivered in the non-late preterm period (*n* = 3,223,777), we identified women who delivered in the late preterm period from 34/0 to 36/6 based on the NHSP-IC database (*n* = 57,963).

Data in relation to women’s health conditions, including obstetric diagnosis, were obtained from the KNHI claims database using International Classification of Diseases-10th Revision (ICD-10) codes. The data on preterm birth and birth weight were checked against the NHSP-IC database. ACS administration and ACS time of administration were confirmed based on the KNHI claims database.

### Study outcomes

Primary short-term outcomes were respiratory complications including transient tachypnea of the newborn (TTN), RDS, and BPD. Secondary short-term outcomes were jaundice, NEC, intraventricular hemorrhage (IVH), and hypoglycemia. Secondary long-term outcomes were neurodevelopmental disorders including autism, cerebral palsy, speech articulation disorder, developmental disorders of scholastic skills, and developmental disorders of motor function.

Using the KNHI claims database, primary and secondary outcomes were identified using primary or secondary diagnoses based on ICD-10 codes. Neonates were classified as having neurodevelopmental disorders if they were diagnosed with neurodevelopmental disorders from birth to December 31, 2018.

### Statistical analysis

Continuous and categorical variables are expressed as mean ± standard deviation and percentages, respectively. Clinical characteristics were compared using analysis of variance for continuous variables and a chi-square test for categorical variables. A multivariable logistic regression analysis model and Cox proportional hazard models were used to estimate the adjusted odds ratios and 95% confidence intervals (CIs) for short-term outcomes and adjusted hazard ratios and 95% CIs for long-term outcomes, respectively. Statistical analyses were performed using SAS for Windows, version 9.4 (SAS Inc., Cary, NC, USA) software. The study protocol was approved by the Institutional Review Board of the Korea University Medical Center.

## Results

Among 57,963 women who gave birth during the late preterm period during the study period, 6,963 (12.1%) had received ACS before 34 weeks ([ACS pre-34 weeks], *n* = 5,708) or after 34 weeks ([ACS post-34 weeks], *n* = 1,255) of gestation.

Table 1 shows study group characteristics according to ACS use and type. Maternal age, birthweight, prevalence of cesarean delivery, and gestational diabetes mellitus differed among a no ACS (no-ACS) group, an ACS pre-34 weeks group, and an ACS post-34 weeks group. The distribution of gestational age at delivery differed among the three groups. We then divided the ACS post-34 weeks group into a dexamethasone use after 34 weeks (dexa post-34 weeks) subgroup and a betamethasone use after 34 weeks (beta post-34 weeks) subgroup. Similar basic characteristics were observed between these four groups.

**Table 1:** Basic characteristics of the study population according to steroid use and types

	No steroid (*n* = 51,000)	Steroid before 34weeks (*n* = 5,708)	Steroid use after 34 weeks			*p*-value	p*-value
			Total (*n* = 1,255)	Dexa use (*n* = 965)	Beta use (*n* = 290)		
Maternal age (years)	31.10 ± 4.11	31.04 ± 3.99	30.75 ± 4.04	30.64 ± 4.01	31.12 ± 4.09	< 0.01	< 0.01
Cesarean deliveries (%)	46.67	40.38	31.87	32.75	28.97	< 0.01	< 0.01
Preeclampsia (%)	11.31	11.02	9.56	9.64	9.31	0.13	0.25
GDM (%)	4.36	5.54	5.18	4.25	8.28	< 0.01	< 0.01
Neonatal male (%)	55.65	55.45	58.09	57.72	59.31	0.21	0.34
Birth weight (kg)	2.49 ± 0.43	2.46 ± 0.43	2.46 ± 0.40	2.47 ± 0.39	2.42 ± 0.42	< 0.01	< 0.01
GA at delivery (%)						< 0.01	< 0.01
34 weeks	20.16	35.13	24.14	23.94	24.83		
35 weeks	38.22	36.72	41.83	42.07	41.03		
36 weeks	41.62	28.15	34.02	33.99	34.14		

P-value for no steroid vs. steroid use before 34 weeks vs. total of steroid use after 34 weeks

P*-value for no steroid vs. steroid use before 34 weeks vs. dexa use vs. beta use

**Fig. 1:**
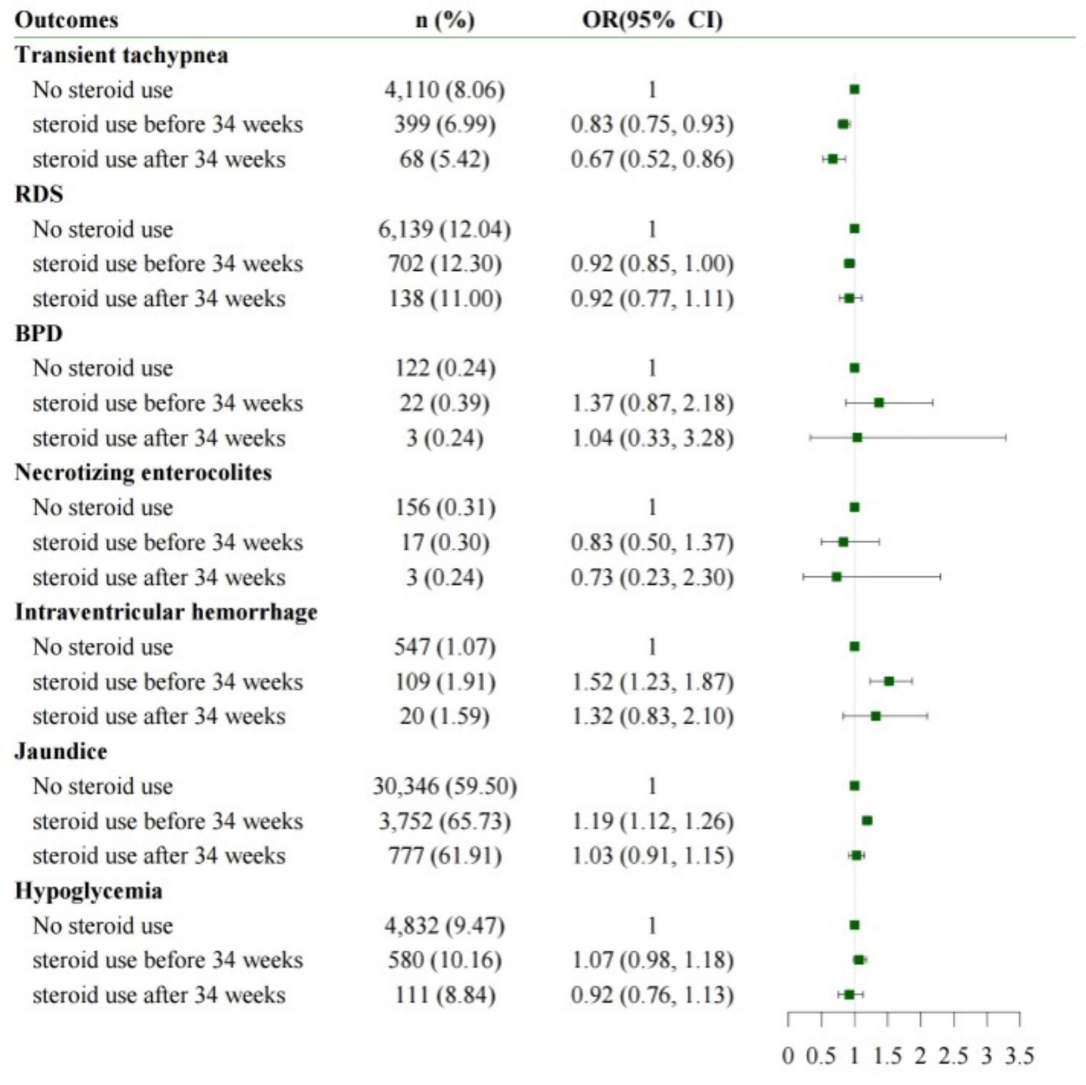
Short-term outcomes according to ACS use ACS, antenatal corticosteroid

Figure 1 shows the short-term outcomes between the three main groups. The ACS post- and pre-34 weeks groups had a lower risk for TTN than the no-ACS group after adjusting for confounding factors, whereas the risk of RDS and BPD did not differ between the three groups. The ACS pre-34 weeks group had a higher risk of jaundice and IVH than the no-ACS group, but no difference was observed in terms of other risks between the ACS post-34 weeks and no-ACS groups.

**Fig. 2:**
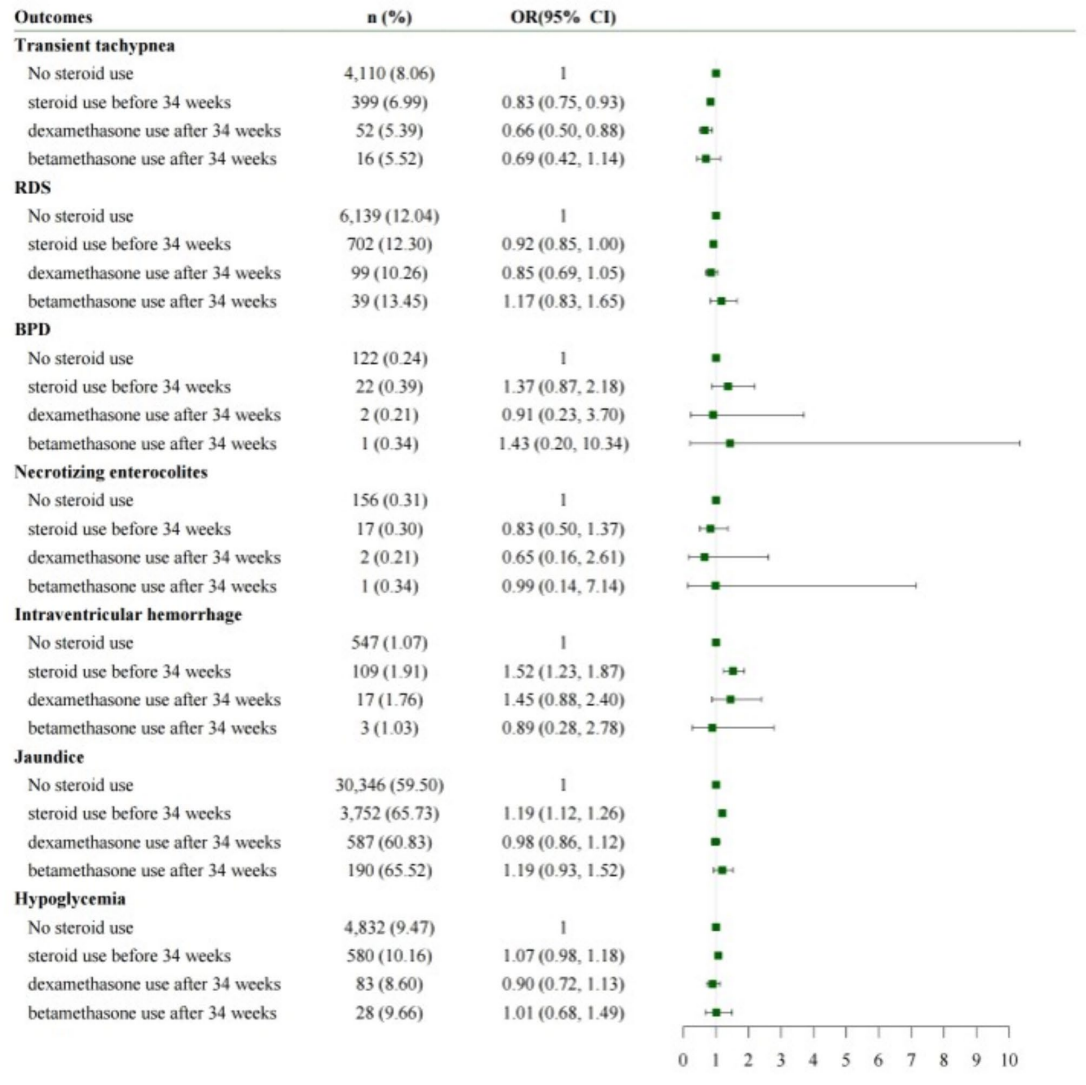
Short-term outcomes according to ACS use and ACS types ACS, antenatal corticosteroid

Figure 2 shows the short-term outcomes after dividing the ACS post-34 weeks group into dexa- and beta post-34 weeks subgroups. The dexa post-34 weeks subgroup had a lower risk of TTN compared with the no-ACS group, but no difference was observed in terms of risk for TTN between the beta post-34 weeks subgroup and the no-ACS group. The dexa- and beta post-34 weeks subgroups did not differ in terms of risk for RDS, BPD, jaundice, NEC, IVH, and hypoglycemia compared with the no-ACS group.

**Fig. 3:**
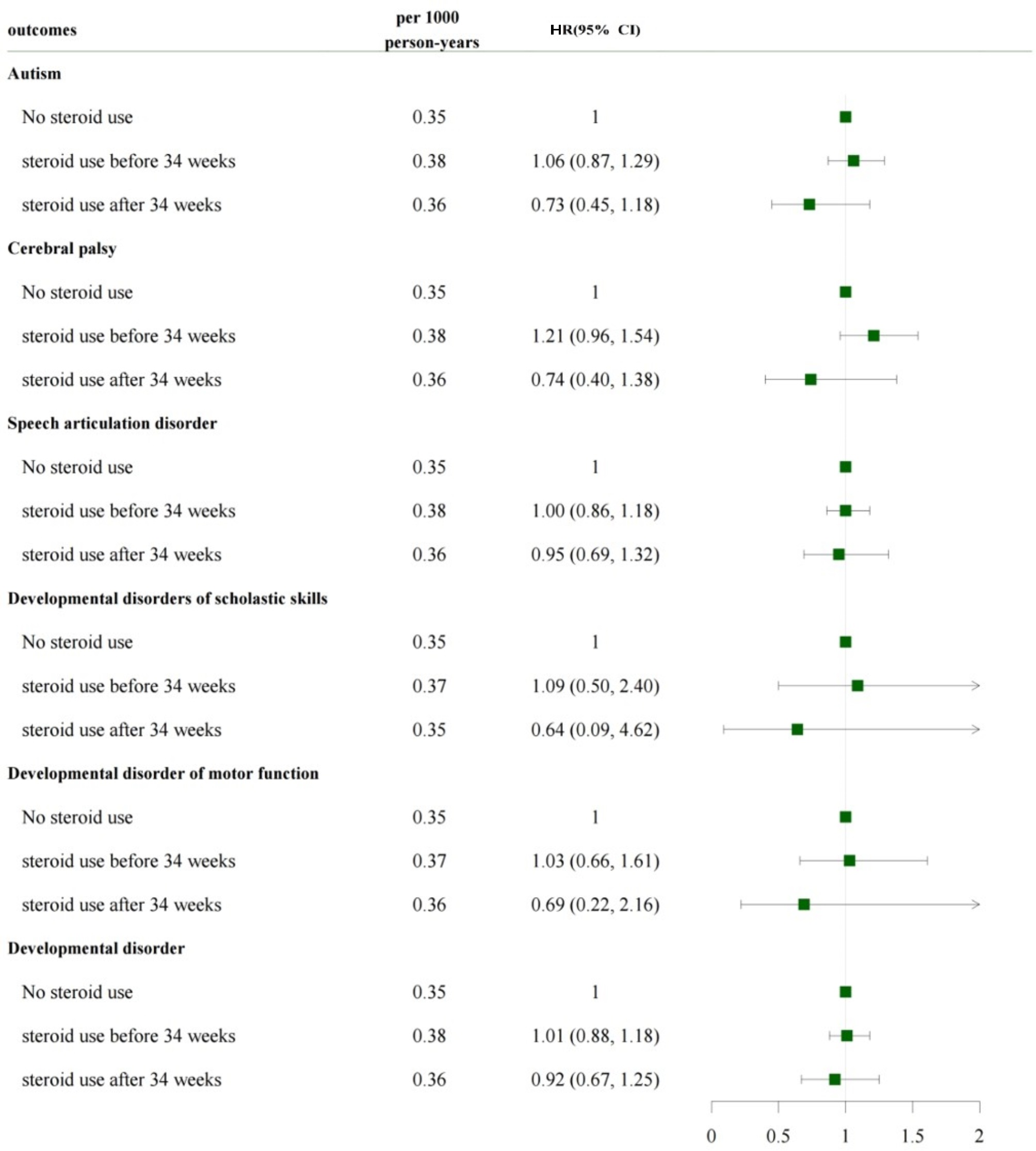
Long-term outcomes according to ACS use ACS, antenatal corticosteroid

**Fig. 4:**
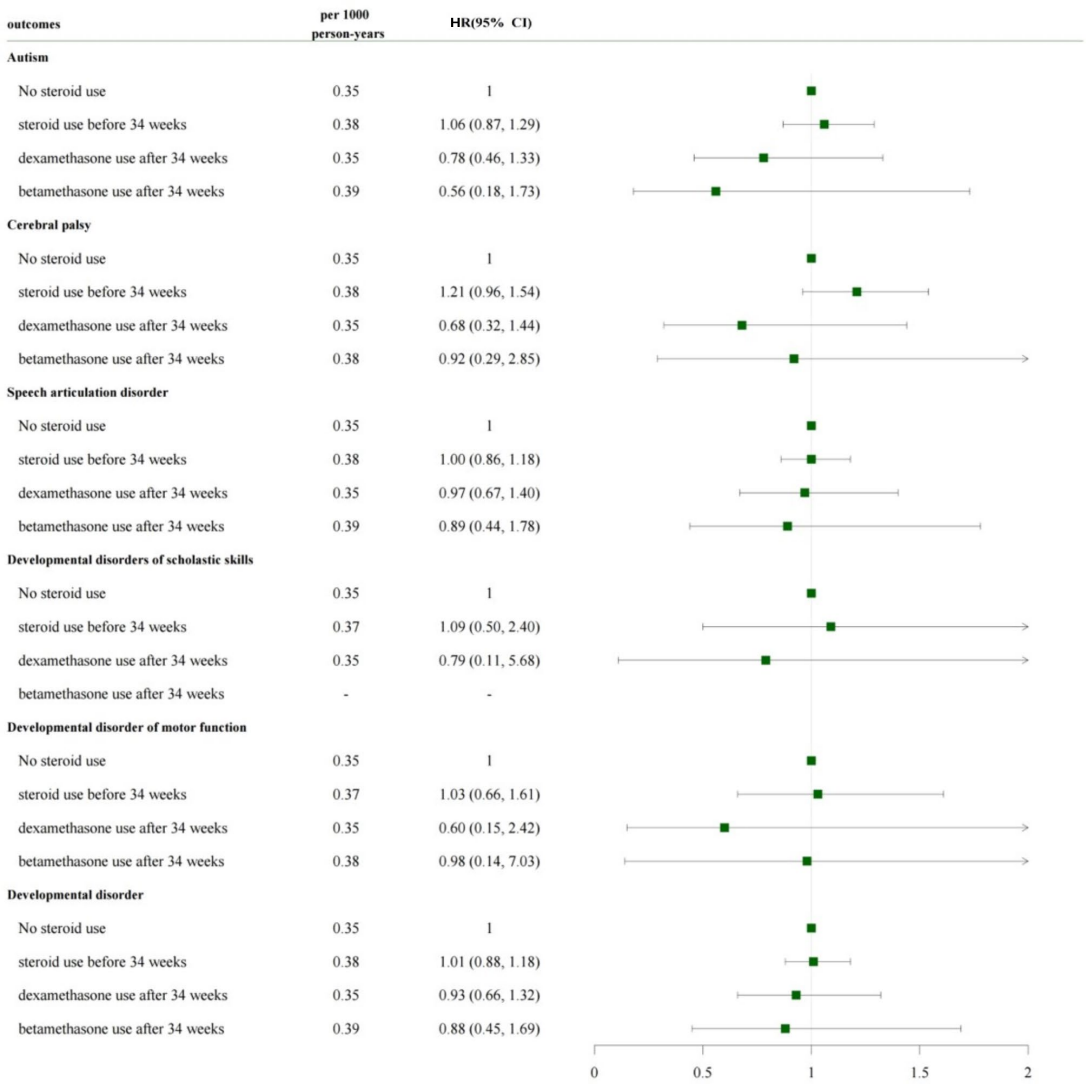
Long-term outcomes according to ACS use and ACS types ACS, antenatal corticosteroid

Figures 3 and 4 show the long-term outcomes between the groups. No difference was observed between the ACS post-34 and ACS pre-34 weeks groups in terms of risk for autism, cerebral palsy, speech articulation disorder, developmental disorders of scholastic skills, developmental disorder of motor function, and developmental disorder, compared with the no-ACS group (Fig. 3). When the ACS post-34 weeks group was divided into dexa- and beta post-34 weeks subgroups, no difference was observed between the subgroups in terms of risk for adverse long-term outcomes compared with the no-ACS group (Fig. 4).

## Discussion

In this study, we evaluated the short- and long-term effects of ACS administration in the late preterm period and compared the effects according to the type of ACS.

Regarding short-term effectiveness for respiratory morbidity, ACS administration in the late preterm term period was associated with a reduction in TTN, suggesting that ACS administration to women at risk for late-preterm delivery reduced the risk of TTN, especially when using dexamethasone, whereas ACS administration in the late preterm period did not affect the risk of other respiratory complications such as RDS and BPD regardless of ACS type. This study is the first to evaluate the long-term effects of ACS administration in the late preterm period on neurodevelopment including cerebral palsy, autism, and development until 11 years of age. Our findings indicated that ACS administration in women who delivered in the late preterm period did not affect neurodevelopment in neonates in relation to autism, cerebral palsy, and developmental disorders, regardless of ACS type.

### Comparison with other studies

Late preterm delivery comprises 71% of preterm births and 8.7% of all live births [22]. Only a limited number of randomized controlled trials (RCTs) have evaluated the effectiveness of ACS administration in the late preterm period, and these trials have reported varied outcomes. In studies concerning betamethasone administration, Porto et al. reported that an administration of one course of betamethasone showed no significant difference in the risk of respiratory morbidity, mechanical ventilation, RDS, or NICU admission [11]. Balci et al. reported that a single dose of betamethasone was associated with a decrease in the need for ventilation and resuscitation, and that betamethasone administration was not associated with RDS [12]. Gyamfi-Bannerman et al., in the ALPS trial, reported that ACS required less surfactant use, and that patients were less likely to require resuscitation or develop BPD and TTN; however, no difference in the risk of RDS, apnea, or the need for mechanical ventilation was observed [13]. In studies concerning dexamethasone, Ontela et al. reported that dexamethasone administration in the late preterm period did not reduce respiratory morbidity (TTN and RDS) [16]. In two studies by Attawattanakul et al., [19, 20] the rate of neonatal respiratory distress was reported to be significantly lower in the dexamethasone group, whereas a requirement for respiratory support and TTN did not differ between the groups [19]. Waiketkarn et al. reported that administration of a single dose of dexamethasone was associated with a reduction in respiratory complications in the late preterm period [20]. 

These discrepancies in the results among the studies may be explained as follows. The outcome variables for respiratory morbidity identified in each study were heterogeneous, and various definitions for the diagnosis of respiratory morbidity were also adopted. As a result, various rates for the same respiratory complication were reported between studies. Moreover, the pregnancy and delivery variables in each study varied. The indications for preterm delivery and delivery mode in each study, which are known to be risk factors for respiratory complications, also differed [23]. Finally, one review reported that more than two-thirds of newborn respiratory complications among late preterm deliveries occurred in those delivering at 34–35 weeks [24], suggesting that ACS was associated with a significant reduction in RDS in babies born between 33 and 34 + 6 weeks of gestation, but that there was no significant reduction in babies born between 35 and 36 + 6 weeks of gestation [1]. Thus, the difference in the distribution of the weeks of gestation in which the patients were enrolled may have been the cause for the discrepancies in the results among these studies. Moreover, in other studies, 10–16% of patients who received ACS in the late preterm period delivered at term [11, 13], but all patients included in this study delivered in the late preterm period. Therefore, it is difficult to directly compare our results with those of other studies.

In this study, there was no significant improvement of RDS in ACS administration before 34 weeks although there was a tendency but the 95%CI was including exactly 1.00. As the effect of ACS will decrease over time, no significant improvement of RDS in women with ACS administration before 34 weeks may be due to the long steroid-to-delivery interval. There are inconsistent and limited data on the association between ACS administration and neurodevelopment in neonates. A meta-analysis reported no evidence of long-term harm to neurodevelopment, particularly when a single course of ACS was administered at < 34 0/7 weeks of gestation [2, 25]. However, a follow-up to a trial of ACS administration at term showed a lower quartile of academic ability as recorded at school [26]. The only data relating to approximately 30-year long-term neurocognitive outcomes after late preterm administration of ACS indicate no effect of betamethasone on cognitive functioning or psychiatric morbidity. However, those at risk of preterm delivery received betamethasone from 30 + 6 to 34 + 4 weeks of gestation and delivered at a median of 35 weeks of gestation (range, 33 + 2–38 weeks of gestation), with 34% of women in the cohort having delivered at term [27]. Based on the findings of our population-based study, while concerns relating to neurodevelopmental harm do not lead us to caution against ACS administration in late preterm, our findings need to be validated in future studies.

### Other outcomes

In the ALPS trial, women who had received ACS at < 34 weeks were excluded. Thus, ACS administration in the late preterm period has not been adequately investigated for all women at risk of late preterm delivery [13]. To address this gap, we also evaluated the effect of ACS administration in women between 24 and 33 weeks of gestation on the incidence of respiratory disorders in neonates born between 34 and 36 weeks of gestation. Our findings showed a reduced rate of TTN, which is consistent with findings reported elsewhere [28]. While the beneficial effects of ACS administration are known to last for at least 1 week, the duration beyond 1 week in terms of these beneficial effects with a single course of ACS is unknown [29]. However, the risk of IVH increased unexpectedly in ACS administration between 24 and 33 weeks of gestation. The precise reason for this finding remains unclear, but it appeared to be in relation to delayed delivery in women with high-risk pregnancies than in those who received ACSs. Further studies are needed to facilitate understanding concerning this finding.

Neonatal hypoglycemia has been associated with impaired neurological outcomes in childhood [30]. The ALPS trial reported a higher incidence of hypoglycemia in an ACS administration group than in a placebo group (25% vs. 14%, respectively) [13], which raised concerns that ACSs may have a long-term detrimental effect on neurodevelopment [17]. However, in our study, the incidence of hypoglycemia did not differ between the groups, which is consistent with previously reported findings [11, 20]. It has been suggested that an increased rate of hypoglycemia in the betamethasone group in the ALPS trail could reflect gestational age, birthweight, or feeding regimens rather than the use of ACS treatment [13]. Moreover, Gyamfi-Bannerman et al., in the ALPS trial, reported that infants with hypoglycemia had shorter median stays in special care nurseries, suggesting that hypoglycemia resolved quickly and was benign [13]. Thus, further studies are needed to assess the incidence of persistent, prolonged hypoglycemia, which is associated with adverse neurologic outcomes [31, 32], rather than the total incidence of hypoglycemia and its association with ACS administration in the late preterm period.

Few studies have evaluated the effectiveness of dexamethasone administration in the late preterm period, with three reporting that it reduced the rate of neonatal respiratory complications [19–21], while one study reported that antenatal dexamethasone did not reduce the rate of respiratory complications; [16] thus, findings have been inconclusive. However, these small sample studies only compared short-term outcomes of respiratory complications between cases with and without dexamethasone. In this study, we compared the short- and long-term effects on neonates born in late preterm period between antenatal use of betamethasone and dexamethasone. Our result suggests that dexamethasone as well as betamethasone can be safely administered in the late preterm period.

### Strengths and limitations

Our study had some limitations. There may have been ICD-10 miscoding of disease in the hospital records. Therefore, our study, which used a nationwide database based on ICD-10 coding, may have included miscoded data. We could not validate the diagnosis of short-and long-term morbidity of neonates by reviewing the medical records. However, all ICD codes in hospitalized patients are strictly reviewed by the Health Insurance and Assessment Service in Korea. Moreover, the accuracy of the diagnostic codes tends to be higher for claims with more severe conditions [33], which would likely include diagnoses in relation to neonates born in the late preterm period.

Our study was also limited in that there was a lack of information regarding the ACS administration complement before delivery and the time between initial dose and date of due delivery, which is a standard limitation in studies using a claims database. While there are limited data on the effects on neonates of an incomplete course of ACSs, it has been reported that incomplete ACS courses are also beneficial for women at < 34 weeks of gestation [34, 35]. The administration of a single dose of ACS to pregnant women in their 34–36th week of gestation, who are likely to have preterm delivery, it reported to reduce RDS development [12, 20]. In the ALPS trial, a benefit was identified despite only 60% of the enrolled women having received a full course of two doses of betamethasone before delivery [13]. Owing to our study’s non-RCT research design and because only data derived from in-hospital records were obtained, it is possible that a certain number of undetected patients with milder forms of neurodevelopmental disorders were missed. The NSHP-IC provides the K-ASQ to screen for developmental delays. Children suspected of having developmental delay on the K-ASQ questionnaire are referred to specialized clinics.^38^ We only included neonates who had been screened, and this involved screening for developmental disorders. The basic characteristics may have differed between the groups because the decision whether to use ACSs in the late preterm period and which ACS to administer was made by the attending obstetrician. Therefore, this study was limited in terms of its ability to control for the participants characteristics owing to its retrospective design.

Nevertheless, a strength of this study was its use of a large population-based cohort with complete and short- and long-term follow-up data. Moreover, owing to the study’s design and data acquisition, no neonate was lost to follow-up.

## Conclusions

Our study findings indicated that ACS administration in the late term period was associated with a reduction in TTN but not with other respiratory complications, including RDS and BPD, and did not affect the neurodevelopment of neonates regardless of the type of ACS used. In this retrospective study, we evaluated the short- and long-term effects of ACS administration in the late preterm period and compared the effects according to the type of ACS. Further trial studies or prospective studies are needed to validate our findings concerning ACS administration in the late preterm period.

## Data Availability

The data that support the findings of this study are available from the National Health Insurance Service (NHIS); however, restrictions apply to the availability of these data, which were used under license for the current study and are, therefore, not publicly available. Data are however available from the authors upon reasonable request and with permission of the NHIS. The results do not necessarily represent the opinion of the National Health Insurance Corporation.

## Abbreviations

ACS: Antenatal corticosteroid
ALPS: Antenatal Late Preterm Steroids
BPD: Bronchopulmonary dysplasia
CI: Confidence interval
HR: Hazard ratio
IVH: Intraventricular hemorrhage
K-ASQ: Korean Ages and Stages Questionnaire
KNHI: Korea National Health Insurance
NEC: Necrotizing enterocolitis
NHSP-IC: National Health-Screening Program for Infants and Children
NICU: Neonatal intensive care unit
RCT: Randomized controlled trials
RDS: Respiratory distress syndrome
